# Application of Unsupervised Machine Learning for the Evaluation of Aerogels’ Efficiency towards Ion Removal—A Principal Component Analysis (PCA) Approach

**DOI:** 10.3390/gels9040304

**Published:** 2023-04-04

**Authors:** Khaled Younes, Yahya Kharboutly, Mayssara Antar, Hamdi Chaouk, Emil Obeid, Omar Mouhtady, Mahmoud Abu-samha, Jalal Halwani, Nimer Murshid

**Affiliations:** 1 College of Engineering and Technology, American University of the Middle East, Egaila 54200, Kuwait; 2 Water and Environment Sciences Lab, Lebanese University, Tripoli 22100, Lebanon

**Keywords:** ion removal, machine learning, aerogels, principal component analysis, water treatment

## Abstract

Water scarcity is a global problem affecting millions of people. It can lead to severe economic, social, and environmental consequences. It can also have several impacts on agriculture, industry, and households, leading to a decrease in human quality of life. To address water scarcity, governments, communities, and individuals must work in synergy for the sake of water resources conservation and the implementation of sustainable water management practices. Following this urge, the enhancement of water treatment processes and the development of novel ones is a must. Here, we have investigated the potential of the applicability of “Green Aerogels” in water treatment’s ion removal section. Three families of aerogels originating from nanocellulose (NC), chitosan (CS), and graphene (G) are investigated. In order to reveal the difference between aerogel samples in-hand, a “Principal Component Analysis” (PCA) has been performed on the physical/chemical properties of aerogels, from one side, and the adsorption features, from another side. Several approaches and data pre-treatments have been considered to overcome any bias of the statistical method. Following the different followed approaches, the aerogel samples were located in the center of the biplot and were surrounded by different physical/chemical and adsorption properties. This would probably indicate a similar efficiency in the ion removal of the aerogels in-hand, whether they were nanocellulose-based, chitosan-based, or even graphene-based. In brief, PCA has shown a similar efficiency of all the investigated aerogels towards ion removal. The advantage of this method is its capacity to engage and seek similarities/dissimilarities between multiple factors, with the elimination of the shortcomings for the tedious and time-consuming bidimensional data visualization.

## 1. Introduction

Water scarcity is a growing problem worldwide, affecting millions of people and leading to various social, economic, and environmental challenges. According to the United Nations, over 2 billion people currently live in countries experiencing high water stress, and it is projected that, by 2050, at least one in four people will be affected by recurring water shortages [1,2]. The causes of water scarcity are complex and varied and include factors such as population growth, climate change, the over-extraction of water resources, poor water management practices, and pollution. These factors have led to a decline in the availability and quality of water in many parts of the world, particularly in developing countries. Water scarcity has a serious impact on human health, agricultural production, industrial development, and environmental sustainability. In areas affected by water scarcity, people often have limited access to clean water, which can lead to waterborne diseases and other health problems [1,2]. A lack of water can also reduce crop yields, which in turn can lead to food shortages and economic instability. To address water scarcity, governments, organizations, and individuals need to work together to promote sustainable water management practices, improve water infrastructure, and reduce water waste and pollution. This includes promoting education and awareness about the importance of water conservation, improving water storage and distribution systems, and investing in water-efficient technologies [1,2].

There are several water purification techniques available based on the difference between the physical/chemical properties of water and the target contaminant [3,4]. Sedimentation and filtration are processes that involve removing large particles and sediment from water through a series of filters and screens. The water is then passed through a fine filter to remove smaller particles. Disinfection is a process that involves using chemicals, such as chlorine, ozone, or ultraviolet light, to kill bacteria and other microorganisms in water [3,4]. Membrane-based water treatment technologies encompass several approaches, depending on the physical phenomenon that describes the concept of the behavior. Reverse osmosis is a water purification technique that uses a semi-permeable membrane to remove impurities from water. It can remove salt, minerals, and other contaminants [3,4]. Ion exchange involves exchanging ions in water with ions of the same charge for the sake of impurities’ removal. Activated carbon filtration involves passing water through activated carbon, which can remove impurities, such as chlorine, volatile organic compounds, and some pesticides. The most effective water purification technique depends on the specific type of contaminants present in the water and the desired end use of the purified water. In many cases, a combination of different techniques may be used to achieve optimal results [3,4].

Adsorption and size exclusion are two important processes used to remove impurities from water. The former involves attracting them to the surface of an adsorbent material, such as activated carbon. The latter involves removing impurities from water based on their size [5,6]. This is achieved through the use of a filter that has small pores, which allow water molecules to pass through while trapping (excluding) larger particles and impurities. Size exclusion is particularly effective at removing suspended solids, such as sediment and algae [5,6]. One of the innovative, green, membrane-based water treatment technologies is the application of aerogels. It represents a class of lightweight and highly porous materials that are often referred to as “frozen smoke” due to their translucent appearance and low density. They are made by removing the liquid component of a gel using a process called supercritical drying, which involves heating the gel under high pressure until the liquid evaporates, leaving behind a solid material with a high surface area and low density [5,6]. Aerogels can be made from a variety of materials, including silica, carbon, and metal oxides. Silica aerogels are the most common type and are often used as insulation in aerospace, construction, and industrial applications due to their high thermal insulation properties. Aerogels have several unique properties that make them attractive for various applications. Their high surface-area-to-volume ratio makes them effective at adsorbing and filtering substances such as oil, heavy metals, and pollutants from air and water [5,6,7]. Their high surface area makes them suitable for use in supercapacitors, batteries, and energy storage and catalytic applications. Additionally, aerogels are promising materials for a range of biomedical applications, including drug delivery, tissue engineering, and biosensors [8,9,10]. Due to their high porosity and adjustable surface chemistry, they can selectively adsorb several water pollutants such as heavy metal ions [11]. Aerogels that contain electron donor elements, such as O, S, and N, are able to form complexes by coordination with the heavy metal ions. The presence of several functional groups, such as hydroxyl, carbonyl, and carboxyl, are also bonded with the metal ions from wastewater [12,13,14,15].

However, aerogels are brittle and fragile, which limits their use in some applications. They are also relatively expensive to produce, which has limited their adoption. In order to overcome the aforementioned problems, continuous investigations of potential aerogels derived from biomass are under consideration. These candidates encompass the “Green Aerogels”. The most common ones under development are the nanocellulose (NC)-based, chitosan (CS)-based, and graphene (G)-based aerogels [7]. The development of such materials requires the synergetic integration of several intercorrelated variables, going from the physical and chemical properties to the adsorption capacities, and even the trade-offs of the manufacturing process and the water treatment conditions’ procedure [5,6,7].

Following the occurrence of multiple source constraints, such as the aforementioned variables involved in aerogels’ fabrication, and to seek merging them in the most systematic way with the least decision errors, a multidimensional statistical analysis method could be adopted. Principal Component Analysis (PCA) is an unsupervised machine learning technique that could be employed to fulfill the constraints. PCA is a statistical technique used to identify patterns and relationships in large datasets [16]. It is a widely used method for data and dimensionality reduction, which involves reducing the number of variables in a dataset while retaining as much of the original information as possible. PCA works by creating new variables, called principal components (PCs), which are linear combinations of the original variables in the dataset [8]. The first PC is the linear combination that accounts for the largest amount of variation in the data. Subsequent PCs are created in order, with each component accounting for as much of the remaining variance as possible. By analyzing PCs, it can be possible to identify patterns and relationships in the data that may not be apparent from the original variables, when a single-handed approach is taken into consideration. It can also be used to identify outliers and to identify which variables are the most important in explaining the variation along the data [16].

In this study, the aim is to apply PCA for the investigation of the relevance of physical/chemical properties and adsorption features towards a set of NC-, CS-, and G-based aerogels. The different data have been obtained from the already published investigations of Paul and Ahankari [7].

## 2. Results and Discussion

PCA analysis was conducted and plotted based on previously published data (Table 1) from the study of Paul and Ahankari [7].

**Figure 1:**
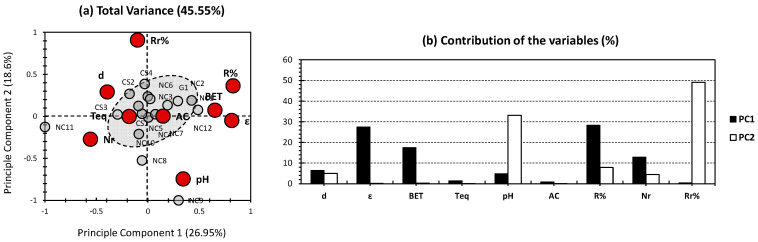
(**a**) PCA biplot for whole dataset. Gray bullets represent the individuals of the population (different investigated NC-, CS-, and G-based aerogels). Red bullets represent the variables (different employed physical/chemical and adsorption parameters). (**b**) Contribution of the variables.

Figure 1 shows the PCA results for the previously published results of the physical/chemical properties and adsorption parameters of several nanocellulose (NC)-, chitosan (CS)-, and graphene (G)-based aerogels implicated in water treatment, from Paul and Ahankari [7]. The first two PCs revealed 45.55% of the total variance (26.95% and 18.60% from the sides of PC1 and PC2, respectively; Figure 1a). Even if the following variance is considered to be average, the yielded multidimensional investigation could reveal some hidden patterns, in comparison to the conventional bidimensional perspective. The obtained rates of the representativeness of the variance could originate from either one of the following scenarios: (1) low correlation between physical/chemical properties, from one side, and the adsorption features from another side or (2) the presence of other properties that are indispensable to explain the adsorption capacities of the investigated aerogels. The first scenario is to be rejected, since the adsorption capacity of a membrane is the synergetic outcome of the different chemical and physical conditions that not only depends on the structural features and chemical components of the aerogel, but goes beyond to the operational conditions of the treatment process. The second scenario is to be acquired, since multiple physical (tortuosity, permeability, viscosity, molecular diffusivity, etc.) and chemical (molecular weight, % of polar/nonpolar functional groups, H-bonding capacity of the matrix, etc.) are not considered [31].

For the variables, the porosity (ε) and removal efficiency (R%) showed the highest contribution in PC1, accounting for 27.40% and 28.27%, respectively (Figure 1b). Apart from the number of reuse/regeneration (Nr) and BET surface area, the rest of the variables showed a very minor contribution towards PC1 (12.87% for Nr and 17.51% for BET; Figure 1b). For PC2, the highest contribution was scored for removal efficiency after regeneration (Rr%), accounting for 49.09% of PC2′s variance. The second highest contributor towards PC2 is the pH, accounting for 33.14% (Figure 1b). The other variables had negligible variance for this PC. The trends indicate an average representativeness of PC1, for R% and Nr as adsorption features; the latter are more likely to be influenced by porosity (ε) and BET surface area, from the physical and chemical properties’ side. Interestingly, BET and ε are located at the same position as R%, with a high positive influence and a moderate one, along PC1 and PC2, respectively (Figure 1a). As for PC2, it shows a high repetitiveness for removal efficiency after regeneration (Rr%), from the adsorption features’ side, and this outcome is more likely to be influenced by the pH.

**Figure 2:**
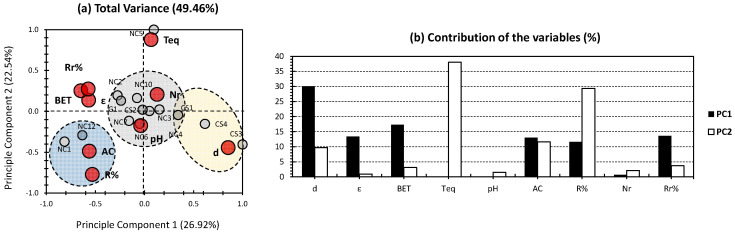
(**a**) PCA biplot for whole dataset, with the exclusion of NC8, NC9, and NC11. Gray bullets represent the individuals of the population (different investigated NC-, CS-, and G-based aerogels). Red bullets represent the variables (different employed physical/chemical and adsorption parameters). (**b**) Contribution of the variables.

**Figure 3:**
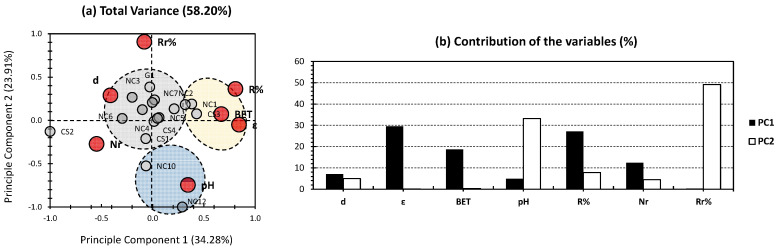
(**a**) PCA biplot for whole dataset, with the exclusion of Teq and AC from the variables. Gray bullets represent the individuals of the population (different investigated NC-, CS-, and G-based aerogels). Red bullets represent the variables (different employed physical/chemical and adsorption parameters). (**b**) Contribution of the variables.

For the individuals, it interestingly showed one bulk clustering of most of the investigated aerogels, around the node (gray cluster, Figure 1a). These trends can give rise to several hypotheses: (1) The individuals of high influence have skewed the different trends of the others; in this case, NC11 has shown a high negative influence, along PC1, with no significant one among PC2. In contrast, NC8 and NC9 have shown moderate and high influences, respectively, along PC2, with no significant one along PC1 (Figure 1a). (2) Several variables showed a low influence for the first two PCs (shown above). In addition, both Teq (time to reach equilibrium) and AC (adsorption capacity) showed a proximity to the node, indicating their low influence towards both PCs (Figure 1a). (3) The investigated aerogels had a high level of similarity in respect to their application in ion removal. This statement cannot be confirmed, due to the moderate total variance yielded in our case. For the sake of validating at least one of the hypotheses, a series of pre-treatments and assumptions will be adopted. For hypothesis (1), an application of PCA to the whole dataset, except for the aerogels with a high contribution, will be taken into consideration (Figure 2). For hypothesis (2), a PCA investigation will be run with the exclusion of Teq and AC (Figure 3). Hypothesis (3) will be supported following the overall trends, yielded by different findings.

Figure 2 shows the PCA findings of the whole dataset, except for NC8, NC9, and NC11. The first two PCs showed a slightly higher total variance of 49.46%, if compared to the one yielded in the case when the whole dataset was taken into consideration (Figure 1). The first two PCs accounted for 26.92% and 22.54%, showing, interestingly, a near influence towards the investigated dataset. These trends allow the attribution of almost equal influences of the first two PCs towards the investigated properties, from one part, and the adopted aerogels, from another part. In addition, a slightly higher dispatchment of the dataset can be noticed, since three clusters were obtained, rather than the one cluster, in the case of the whole-dataset PCA (Figure 1). For the variables, density showed the highest contribution towards PC1, accounting for 30.05% (Figure 2a). Average contributions were yielded for the rest of the variables along PC1. For PC2, time to reach equilibrium (Teq) and removal efficiency (R%) exhibited the highest contributions, accounting for 38% and 29.32%, respectively (Figure 2). Similar to the PCA of Figure 1, two variables were yielded near the node, yet different variables for this case (Nr, and pH; Figure 2). In addition, there was an agglomeration of removal efficiency after regeneration (Rr%), BET surface area, and porosity (ε) were conserved. This trend indicates the similar influence of these variables along the investigated aerogels, in contrast to their lower one on the excluded aerogels (NC8, NC9, and NC11 in this case; Figure 2). When the approach of excluding individuals is adopted, a huge turnover of the different trends can be noticed. The most noticeable ones are the high contribution of Teq, which was negligible previously, and the tremendous decrease in the contribution of removal efficiency after regeneration (Rr%).

For the individuals, three different clusters can be distinguished (gray, yellow, and blue; Figure 2a). For the gray cluster, it showed a centered position around the node and was positively correlated along with the pH and the number of reuse/regeneration (Nr). Interestingly, this cluster has gathered most of the investigated aerogels. For the yellow cluster, it gathered CS3 and CS4, and to a lower extent CS1, and showed a high positive correlation along the most influential variable, the density. CS1 was yielded at the interface between this cluster and the gray cluster. Interestingly, the yellow cluster has gathered all chitosan-based aerogels, except for CS2 (Figure 2a). It showed a moderate-to-high positive correlation among PC1, with a low-to-negligible one along PC2. For the blue cluster, it combined NC1 along with NC12 and showed a high positive correlation along the adsorption capacity (AC) and the removal efficiency (R%). It showed a high and moderate negative correlation, along PC1 and PC2, respectively (Figure 2a). NC5 was located individually on the high positive side of PC2, with a negligible influence along PC1. This aerogel peculiarly showed a high positive effect along the time required to reach equilibrium (Teq; Figure 2a). Following the aforementioned results (Figure 1 and Figure 2), the exclusion of some individuals has shown a slightly higher efficiency in presenting the different trends, in the bidimensional perspective. Nonetheless, the recurrent agglomeration of most of the investigated aerogels around the node makes this method more likely unreliable in this case.

Figure 3 shows the PCA findings of the whole dataset, with the exclusion of time to reach equilibrium (Teq) and adsorption capacity (AC) from the variables. The first two PCs showed 58.20% of the total variance (34.28% and 23.91% for PC1 and PC2, respectively; Figure 3a). This higher value, in comparison with the two previous investigations (Figure 1 and Figure 2), ascertains the efficiency of the adopted approach, towards the reveal of a higher variance in data treatment when variables with minor influence are discarded. For the variables, a profile of contributions similar to the PCA of the whole dataset was noticed (Figure 1b and Figure 3b). These trends make sense, since the variables excluded (Teq and AC; Figure 3) possessed a minor contribution towards the investigated aerogels.

For the individuals, three different clusters can be distinguished (gray, yellow, and blue; Figure 3a). For the gray cluster, it showed a centered position around the node and was positively correlated along with the density, which is considered a minor contributor for both PCs (Figure 3b). For the yellow cluster, it gathered CS3 and NC1 and showed a moderate contribution along PC1. This cluster was positively influenced by removal efficiency (R%), BET surface area, and porosity (ε), which are considered moderate contributors towards PC1 (Figure 3b). For the blue cluster, it gathered NC10 and NC12 and showed moderate-to-high positive correlation along PC2, with a slight negative and positive correlation for PC1 (Figure 3a). It showed a positive influence by the pH, which is considered a moderate contributor towards PC2 (Figure 3b). For CS2, it was exclusively located at the negative SIDE of PC1, with a minor influence by PC2. This aerogel was mostly influenced by the number of reuse/regeneration (Nr).

## 3. Conclusions

In this work, we have envisaged to perform, for the first time, “Principal Component Analysis” (PCA) in the purpose of estimating the efficiency of ion removal along several types of aerogels. The three categories, in-hand, are nanocellulose (NC)-based, chitosan (CS)-based, and graphene (G)-based aerogels. In the case of an all-in-one dataset approach (Figure 1), a moderate total variance has been obtained, indicating a low repetitiveness of the “Total Truth”. From the variables side, an interesting high rate of separation between variables can be noticed, between the first two PCs. In fact, density (d), porosity (ε), and BET surface area are the highest influencers for removal efficiency(R%) and the number of reuse/regeneration (Nr) to a lower extent. Additionally, pH as a physical/chemical property has been found to be the most influential for removal efficiency after regeneration (Rr%). Nonetheless, the aforementioned findings cannot be confirmed, due to the slightly low variance of the first two PCs. In order to overcome this issue, two hypotheses have been investigated, requiring two different approaches. The first hypothesis implies that the high influence of a minority of the investigated samples (NC8, NC9, and NC11, in this case) have biased the whole dataset of the PCA biplot. In order to seek the validity of this statement, a PCA investigation was envisaged, without the consideration of NC8, NC9, and NC11 (Figure 2). The aforementioned approach has slightly raised the total variance, indicating its efficiency towards revealing different aerogel samples, along the adopted properties. The second hypothesis implies that the low influence of some of the variables (AC and Teq, in this case) has skewed the different trends of the multidimensional PCA approach. In order to overcome this issue, a PCA investigation discarding the low impact variables has been adopted (Figure 3). The aforementioned strategy has shown its efficiency in revealing better trends in the investigated dataset, as a more noticeable increase has been scored (around 58% of the total variance; Figure 3). The third hypothesis implied a high similarity of the investigated aerogels in respect to their application in ion removal. The latter hypothesis is the most likely to be true, since when applying both approaches a similar trend was obtained. This is noticeable in the sense that, for the three followed PCA approaches, the different aerogel samples were located in the center of the biplot and were surrounded by different physical/chemical and adsorption properties. This would probably indicate the similar efficiency in ion removal of the aerogels in-hand, whether they were nanocellulose-based, chitosan-based, or even graphene-based.

## 4. Materials and Methods

The methodology approach in this work is similar to our previously published works in the application of PCA [32,33]. PCA is a statistical technique that simplifies complex datasets by reducing the number of variables while retaining the most important information. It achieves this by transforming the original variables into a smaller set of uncorrelated variables, known as principal components (PCs), through linear combinations. The first PC captures the direction of the highest variability in the dataset, with subsequent PCs being orthogonal to previous components and capturing the next highest variability. PCA is commonly used in data analysis and machine learning to extract valuable insights from large datasets, identify patterns and relationships, and detect outliers and anomalies. However, its effectiveness is limited in cases where nonlinear relationships or complex structures exist, and it can be sensitive to outliers [16].

### 4.1. Data Collection and Pre-Treatment

Data have been collected from the published study of Paul and Ahankari [7]. Table 1 presents the inventory of the different investigated NC-, CS-, and G-based aerogels, along their performance capacity, adsorption parameters, and physical/chemical characteristics.

The data of each of the investigated variables have different weights. To remove any bias yielded by the difference of magnitude, a normalization technique, such as the one of Younes et al. (cite), has been adopted as follows:

(1) $$Y_{st}=\frac{\left(Value-Mean\right)}{Standard\;Deviation}$$

where “*Y_st_*” presents the standardized dataset values.

### 4.2. Principal Component Analysis (PCA)

After normalization, PCA findings were yielded using XLSTAT 2014 software, following an approach similar to the one adopted by Murshid et al. [17]. In this study, the missing data were estimated using a built-in feature that replaces a missing value with the “Mode”, following the respective variables.

The aim of this study is to apply PCA on the data found in a previous study by Paul and Ahankari [7] (Table 1), applying PCA targets searching for any hidden layers between the physical/chemical properties, from one side, and adsorption parameters, from another side. In the case that they are found, this will help in the better interpretation, and therefore better understanding, of different factors that influence the applicability of a certain aerogel membranes. The output information yielded by PCA could help in several stages of the water treatment process, from the manufacturing approach going to the experimental conditions to the removal efficiency of a selected membrane. Here, we have applied PCA, for 8 different factors, influencing 24 investigated aerogels (Table 1). PCA is a data-driven unsupervised machine learning technique, which works on the reduction of a certain dataset. The outcome of such reductions has been applied for a better visualization of a certain phenomenon to seek hidden knowledge by the given correlations (negative or positive) and the representativity of the principal components (PCs) to the population in-hand. The jth PC matrix (*Fi*) is expressed using a unit-weighting vector (*Uj*) and the original data matrix *M* with m x n dimensions (m: number variables, n: number of datasets), as follows [34,35,36,37]:

(2) $$Fi=U_{j}^{T}M=\sum^{i=0}U_{ji}M_{i}$$

where *U* is the loading coefficient and *M* is the data vector of size *n*. The variance matrix *M*(*Var*(*M*)) is obtained by projecting *M* to *U* and should be maximized, as follows:

(3) $$Var\left(M\right)=\frac{1}{n}\left(UM\right){\left(UM\right)}^{T}=\frac{1}{n}{UMM}^{T}U$$

(4) $$MaxVar\left(M\right)=Max\left(\left(\frac{1}{n}\right){UMM}^{T}U\right)$$

Since $\frac{1}{n}{MM}^{T}$ is the same as the covariance matrix of *M*(*cov*(*M*)), *Var*(*M*) can be expressed, as follows:

(5) $$Var\left(M\right)=U^{T}cov\left(M\right)U$$

The Lagrangian function can be defined, by performing the Lagrange multiplier method, as follows:

(6) $$L=U^{T}$$

(7) $$L=U^{T}cov\left(M\right)U-\delta (U^{T}U-1)$$

For Equation (7), “*U^T^U* − 1” is considered to be equal to zero, since the weighting vector is a unit vector. Hence, the maximum value of *Var*(*M*) can be calculated by equating the derivative of the Lagrangian function (*L*), in respect to *U*, as follows:

(8) $$\frac{dL}{dU}=0$$

(9) $$cov\left(M\right)U-\delta U=\left(cov\left(M\right)-\delta I\right)U=0$$

where *δ* is the eigenvalue of *cov*(*M*) and *U* is the eigenvector of *cov*(*M*).

## Figures and Tables

**Table 1:** Nanocellulose (NC)-, chitosan (CS)-, and graphene (G) oxide-based aerogels in ion removal: physical/chemical and adsorption parameters. (Adapted from Ref. [7] with permission from Elsevier.).

Sl. No.	Aerogel Composition	Physical/Chemical Parameters					Adsorption Parameters				Ref
		Density (mg/cm^3^)	Porosity (%)	BET Surface Area (m^2^/g)	Time to Reach Equilibrium (min)	pH	Adsorption Capacity (mg/g)	Removal Efficiency (%)	Number of Reuse/Regenerations	Removal Efficiency (%) after Regenerations	
Nanocellulose (NC)-based Aerogels											
1	TCTGAs	7	99.5	-	2	5.5	485.4	100	5	90	[13]
2	TO-CNF-Si-NH_2_	12.4	99	144.3	-	5	99	95.6	-	87.2	[14]
3	PDA-CNF-PEI	25	98.5	-	720	5	103.5	-	4	91	[15]
4	CGP	-	-	-	600	5.6	163.4	-	5	-	[17]
5	U-EDTACCA	5	99	-	7200	5	104	91	5	88	[18]
6	CNF/PEI	-	-	42.5	120	5	357.1	-	3	90	[19]
7	TO-CNF/TMPTAP/PEI	-	-	-	1200	5.5	485.4	-	4	-	[20]
8	BNC/MoS2	-	-	117	120	5.3	-	88	6	10	[21]
9	BHA	8.2	99.4	54	180	10	217	-	5	2	[22]
10	TO-CNF-TMPTAP-APAM	14.4	99.1	-	1560	6	240	-	10	-	[23]
11	BRU/PNFCA	-	91.6	1.03	1380	4.75	332.7	83.4	8	83	[24]
12	MOF/CNC-CMC	8	-	125	2	6	575	97	2	-	[25]
**Chitosan (CS)-based Aerogels**											
1	ZnBDC/CSC	-	-	16.3	25	5	225	94	5	84	[26]
2	CS-PDA	-	-	77.3	96	2	374.5	-	8		[27]
3	CPA	43	-	5.9	360	6	163.7	-	6	70	[28]
4	E-CS	38.3	97.4	-	240	5	108.1	95	3	91	[29]
**Graphene (G)-based Aerogels**											
1	rGO	12.2	-	136.7	50	5.5	58	-	4	90	[30]

## Data Availability

The data presented in this study are available on request from the corresponding author.
